# Randomized clinical trial of astaxanthin supplement on serum inflammatory markers and ER stress‐apoptosis gene expression in PBMCs of women with PCOS

**DOI:** 10.1111/jcmm.18464

**Published:** 2024-07-22

**Authors:** Masoome Jabarpour, Fardin Amidi, Ashraf Aleyasin, Maryam Shabani Nashtaei, Mojtaba Saedi Marghmaleki

**Affiliations:** ^1^ Department of Anatomy, School of Medicine Tehran University of Medical Sciences Tehran Iran; ^2^ Department of Infertility, Shariati Hospital Tehran University of Medical Sciences Tehran Iran; ^3^ Department of Infertility, Yas Hospital Tehran University of Medical Sciences Tehran Iran

**Keywords:** apoptosis, astaxanthin, ER stress, inflammatory markers, PBMC, polycystic ovary syndrome

## Abstract

Polycystic ovarian syndrome (PCOS) is related to pro‐apoptotic and pro‐inflammatory conditions generated by Endoplasmic reticulum (ER) stress. This study aimed to determine the effect of Astaxanthin (ASX), as carotenoid with potent antioxidant and anti‐inflammatory properties, on serum inflammatory markers, apoptotic factors and ER stress‐apoptotic genes in peripheral blood mononuclear cells (PBMCs) of women with PCOS. This randomized, double‐blind clinical trial included 56 PCOS patients aged 18–40. For 8 weeks, subjects were randomly assigned to one of two groups: either 12 mg ASX (*n* = 28) or placebo (*n* = 28). Real‐time PCR was used to quantify gene expression associated with ER stress‐apoptosis in PCOS women's PBMCs. The levels of TNF‐α, IL18, IL6 and CRP were determined by obtaining blood samples from all patients before and after the intervention using Enzyme‐linked immunosorbent assay (ELISA). Also, the levels of active caspase‐3 and caspase‐8 were detected in the PBMC by ELISA kit. Furthermore, we evaluated the efficacy of ASX on disease symptoms. Following the 8‐week intervention, ASX supplementation was able to reduce the expression of GRP78 (*p* = 0.051), CHOP (*p* = 0.008), XBP1 (*p* = 0.002), ATF4 (0.038), ATF6 (0.157) and DR5 (0.016) when compared to the placebo. However, this decrease was not statistically significant for ATF6 (*p* = 0.067) and marginally significant for GRP78 (*p* = 0.051). The levels of TNF‐α (*p* = 0.009), IL‐18 (*p* = 0.003), IL‐6 (*p* = 0.013) and active caspase‐3 (*p* = 0.012) were also statistically significant lower in the therapy group. However, there was no significant difference in CRP (*p* = 0.177) and caspase‐8 (*p* = 0.491) levels between the treatment and control groups. In our study, ASX had no significant positive effect on BMI, hirsutism, hair loss and regularity of the menstrual cycle. It appears that ASX may benefit PCOS by changing the ER stress‐apoptotic pathway and reducing serum inflammatory markers; however, additional research is required to determine this compound's potential relevance.

## 1 INTRODUCTION

Among women of childbearing age, PCOS is the most common endocrine condition and the major cause of infertility.^1^ There is a wide variation in the prevalence of this syndrome across countries.^2^ It may be affected by factors such as racial diversity and physical composition.^3^ According to the Rotterdam agreement, more than 15% of women have PCOS.^4^, ^5^ There are numerous characteristics associated with PCOS, such as hyperandrogenism (HA), metabolic disturbances, irregular menstruation, anovulation, hirsutism and infertility.^6^ There is no clear explanation for the aetiology of PCOS, but genomic and environmental factors are believed to contribute to this condition.^7^, ^8^ According to recent research, chronic low‐grade inflammation has a significant role in the development of PCOS.^9^ In fact, as a pro‐inflammatory condition, PCOS is associated with cardiovascular disease and type 2 diabetes.^10^ In numerous investigations comparing women with this syndrome to controls, tumour necrosis factor (TNF‐α), interleukin‐6 (IL‐6) and interleukin‐18 (IL‐18), as well as high‐sensitivity C‐reactive protein (hs‐CRP), were shown to be significantly elevated.^11^, ^12^ It was observed that there is a positive correlation between TNF‐α and IR. Additionally, IR may increase the level of IL‐6, a known risk factor for cardiovascular disease in females. There is also a correlation between increased levels of IL‐1α, IL‐1β, IL‐2, IL‐8, IL‐9, IL‐15, IL‐17, IL‐23 and interferon‐γ (IFN‐γ) with PCOS. Researchers hypothesize that hyperandrogenism in PCOS may have stimulated the local macrophages, thereby contributing to the pro‐inflammatory milieu.^13^, ^14^, ^15^, ^16^ In addition, numerous studies have demonstrated the involvement of apoptotic dysregulation in the development of PCOS. Caspases play a critical role in the process of apoptosis. There is evidence suggesting that caspases 1, 3, 7, 8 and 9 are involved in the development of PCOS.^17^, ^18^, ^19^ Studies have also looked at the connection between PCOS and Endoplasmic reticulum (ER) stress. ER stress has been found to be higher in PCOS/obese PCOS patients as well as mouse models for PCOS compared to non‐PCOS controls in previous investigations.^20^, ^21^, ^22^ ER stress develops as a pathologic condition when unfolded or misfolded proteins accumulate in tissues with excessive protein production. The induction of ER stress can occur under physiological and pathological circumstances such as glucose deprivation, inflammation and oxidative stress (OS), as well as high free fatty acid levels, abnormal Ca^2+^ regulation and hypoxia.^23^ Under ER stress, unfolded protein response (UPR) occurs to protect the cells.^23^ UPR is regulated by the 78‐kDa glucose‐regulated protein (GRP78) and the three transmembrane proteins it contains: protein kinase R (PKR)‐like endoplasmic reticulum kinase (PERK), inositol‐requiring enzyme 1 (IRE1) and activating transcription factor 6 (ATF6).^24^ If ER stress cannot be alleviated, the cell undergoes apoptosis.^25^ It's still not clear how ER stress triggers apoptosis, and the mechanism may vary depending on the stimulus and cell type. Cell apoptosis under ER stress is clearly linked to overexpression of the UPR transcription factor C/EBP homologous protein (CHOP).^25^ In apoptosis brought on by ER stress, the CHOP‐targeted death receptor 5 (DR5) plays an important role by boosting the generation of the autocrine death ligands signal, which in turn causes more cell death.^26^, ^27^, ^28^ A recent study has demonstrated that human and mouse cumulus cells undergo apoptosis after testosterone‐induced ER stress, via induction of DR5.^29^ A number of studies have suggested that DRs and caspase‐8 may contribute to the promotion of apoptosis subsequent to ER stress.^26^, ^27^, ^30^, ^31^ It has been shown that, caspase‐8 has the ability to initiate the activation of caspase‐3 and caspase‐7.^32^ ER stress, on the other hand, exerts a close relationship with inflammation and OS. It has been demonstrated that all three main UPR pathways regulate the transcriptional program that promotes inflammation through transcription factors like NF‐ΚB (Nuclear factor‐κB) and activator protein‐1 (AP‐1).^33^

PCOS is often associated with inflammation, obesity, insulin resistance (IR) and HA, which are all common symptoms and potential causes. These factors have a strong correlation with OS. In addition, excessive androgen levels can lead to OS, IR and inflammation. The interaction of these factors contributes to the development and worsening of ovulation disorders in PCOS, particularly in individuals who are overweight or obese.^34^, ^35^ Recently, there has been increasing emphasis on the use of antioxidants as an adjuvant treatment for PCOS. It has been reported that ASX, as a xanthophyll carotenoid found in several microorganisms, has been reported to be a potent antioxidant with no negative effects. It has been found that ASX has anti‐inflammatory, antiapoptotic, antioxidative, anticancer, neuroprotective and immunomodulatory effects, among others.^36^ According to considerable in vivo and in vitro research, ASX appears to have anti‐inflammatory properties in mammals, raising the prospect that it could be a useful treatment for disorders related to inflammation.^37^ Moreover, prior investigations have shown that ASX suppresses ER stress.^38^, ^39^, ^40^ Meanwhile, recent research from our lab suggested that ASX may be able to influence ER stress and OS in the granulosa cells of PCOS patients.^41^ As OS induces ER stress and plays a pivotal role in the pathogenesis of PCOS, and also considering the interactions between ER stress, inflammation, OS and apoptosis, in this study, we examined the effect of ASX on serum inflammatory markers, active caspase levels and gene expression related to ER stress‐ apoptosis in PBMC of women with PCOS. Furthermore, we evaluated the efficacy of ASX on disease symptoms.

## 2 METHODS

### 2.1 Trial design and participants

Between October 2021 and May 2022, 56 PCOS patients aged 18–40, were enrolled in the current randomized, double‐blind trial at the Omid Clinic in Tehran, Iran, which is registered at http://www.irct.ir: IRCT20201029049183N2. Prior to any intervention, all participants signed informed consent forms (ethics committee reference number: IR.TUMS.MEDICINE.REC.1400.1051). Pregnant women and women with metabolic disorders, including thyroid disease, Cushing's syndrome, hyperprolactinemia, diabetes mellitus, congenital adrenal hyperplasia and impaired glucose tolerance, as well as current or particular past diet or physical activity programs (within the last 3 months) were not included in the study. In addition, all of the patients in our research had no medical history of inflammatory or autoimmune diseases. For 8 weeks, participants were randomized into two groups and given either a placebo (*n* = 28) or 12 mg capsules in terms of colour, shape, size, packaging and other attributes. Astareal Company (Tokyo, Japan) provided ASX capsules and placebos. Checking the capsule containers and sending daily text messages reminding participants to take their supplements and placebos were used to gauge compliance. The compliance was ensured through the use of 24‐h food diary records completed during the study. Additionally, a modified version of the Nutritionist IV program, which is based on Iranian foods, was utilized to estimate the dietary intake of patients. In addition, we utilized the Persian version of the 7‐item International Physical Activity Questionnaire (IPAQ) to monitor and regulate physical activity levels. The scoring system provided by the IPAQ was used to classify physical activity levels into three categories: inactive, minimally active and health‐enhancing physically active. Regarding clinical signs, a 21–35‐day menstrual cycle was considered regular. The study also used the Ferriman‐Gallwey criteria to determine the hirsutism score, as well as the Ludwig visual score for hair loss.

### 2.2 Assessment of outcomes

Inflammatory markers were considered as the primary outcomes, and biomarkers of apoptosis, gene expression, clinical features of hyperandrogenism (hirsutism and hair loss), and irregular menstruation cycle were considered as the secondary outcomes.

### 2.3 Randomization and blinding

**FIGURE 1:**
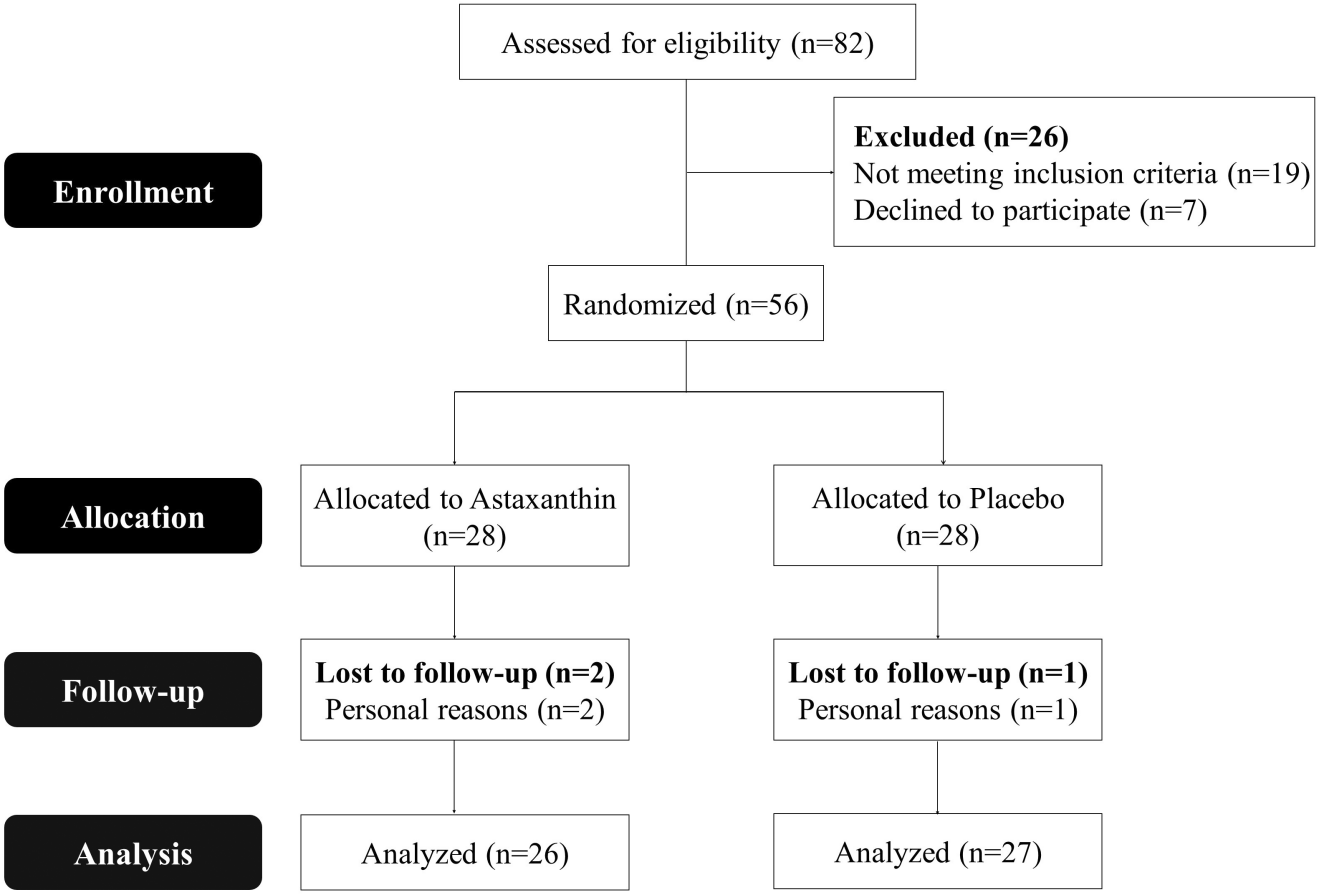
Flow diagram of patients' recruitment.

The study began with the inclusion of 56 women. We used blocked randomization to assign participants to either a control (placebo) or intervention (ASX) group at random. Participants and researchers were kept in the dark about randomization and allocation to the study. An overview of the study map can be seen in Figure 1.

### 2.4 Isolation of lymphocyte cells

Density gradient centrifugation with Ficoll–Hypaque (Lymphodex, Inno‐Train, Germany) was used to isolate lymphocytes from the blood. It is best to describe the procedure as follows: fresh blood is combined with an equal volume of Phosphate Buffer Saline (PBS), and then carefully layered over Ficoll in a 2:1 ratio. The sample was subjected to 1100 g of centrifugal force for 25 min at 20°C. As a final step, the middle layer was slowly transferred to a fresh falcon and twice washed with PBS at 300 g for 10 min.

### 2.5 RNA extraction and real‐time PCR

The extraction of RNA was done manually. The RNX‐plus Solution was utilized in the process of RNA isolation (Cinnacolon, Tehran, Iran). Until cDNA could be made, it was held at −20°C. A spectrophotometer was used to determine the quantity of total RNA extracted from each sample (Biochrom WPA Biowave, Cambridge, UK). Then, we synthesized complementary DNA, using a cDNA synthesis kit (Sinnaclon, Tehran, Iran) according to the manufacturer's instructions. Quantitative RT‐PCR was used to evaluate the expression of XBP1, ATF4, ATF6, GRP78, CHOP, DR5 and glyceraldehyde‐3‐phosphate dehydrogenase (GAPDH) as a housekeeping gene using Lightcycler 96 System (Roche, Germany) and Real Q Plus 2x Master Mix Green (Amplicon, Denmark). mRNA expression calculations were calculated using the Livak technique (2^−ΔΔCt^) (Livak and Schmittgen, 2001). Table 1 displays the primer sequences.

**TABLE 1:** Forward and reverse primers used for real‐time quantitative PCR.

Gene	Primer
GRP78	F: CTGTCCAGGCTGGTGTGCTCT R: CTTGGTAGGCACCACTGTGTTC
CHOP	F: GGTATGAGGACCTGCAAGAGGT R: CTTGTGACCTCTGCTGGTTCTG
ATF4	F: TTCTCCAGCGACAAGGCTAAGG R: CTCCAACATCCAATCTGTCCCG
ATF6	F: CAGACAGTACCAACGCTTATGCC R: GCAGAACTCCAGGTGCTTGAAG
XBP1	F: CTGCCAGAGATCGAAAGAAGGC R: CTCCTGGTTCTCAACTACAAGGC
DR5	F: AGCACTCACTGGAATGACCTCC R: GTGCCTTCTTCGCACTGACACA
GAPDH	F: CGC CAG CCG AGC CAC ATC R: CGC CCA ATA CGA CCA AAT CCG

Abbreviations: ATF4, activating transcription factor4; ATF6, activating transcription factor 4; CHOP CCAAT, enhancer‐binding protein homologous protein; DR5, Death receptor 5; GAPDH, glyceraldehyde‐3‐Phosphate dehydrogenase; GRP78, glucose regulated protein78; XBP1, X‐box binding protein 1.

### 2.6 Enzyme‐linked immunosorbent assay

At the beginning and end of the intervention, 10 mL of venous blood samples were collected to determine serum levels of TNF‐α, IL‐18, IL‐6, CRP, caspase‐3 and caspase‐8. Abcam enzyme‐linked immunosorbent assay (ELISA) kit utilized according to the manufacturer's instructions for TNF‐α, IL‐18 and IL‐6 (abcam ab181421, ab215539 and ab178013, USA, respectively). In addition, CRP was evaluated using the particle enhanced turbid metric approach in this study (Pishtaz‐Teb Diagnostics, Tehran, Iran). Also, we utilized Invitrogen Thermo Fisher Scientific kits (Invitrogen, Camarillo, CA, USA), for detection the levels of caspase‐3 (KHO1091) and caspase‐8 (BMS2024) in the PBMC, in compliance with the manufacturer's protocols. The results were presented per μg of protein found in PBMC lysates.

### 2.7 Statistical analysis

The Kolmogorov–Smirnov test was utilized to confirm the parameters' normality, and data were reported as means± SD (standard deviations). In accordance with the proposed formula for parallel clinical trials (Kelsey et al.), and serum CRP levels as a key variable, we calculated a sample size of 25 participants per each group^42^ based on type I error of 5% (*α* = 0.05), type II error of 20% (*β* = 0.20; power = 80%). Given the anticipated 5% attrition rate, the sample size for each group was determined to be 28 individuals. Statistical analysis was performed using SPSS version 22 with a significance level of *p* < 0.05. In order to compare the baseline characteristics of the two groups, we utilized a chi‐square test for categorical data and an independent sample *t*‐test for continuous data. Also, variables change between the two intervention groups were compared by independent sample *t*‐tests. For intra‐group changes, Mcnemar test used for categorical variable. In order to avoid potential bias, analysis of covariance (ANCOVA) was used to adjust for the effects of baseline values (dependent variable) such as BMI and age. The correlations between the serum levels of the cytokines with the signs of diseases and baseline variables were investigated using Pearson's correlation coefficient.

## 3 RESULTS

**FIGURE 2:**
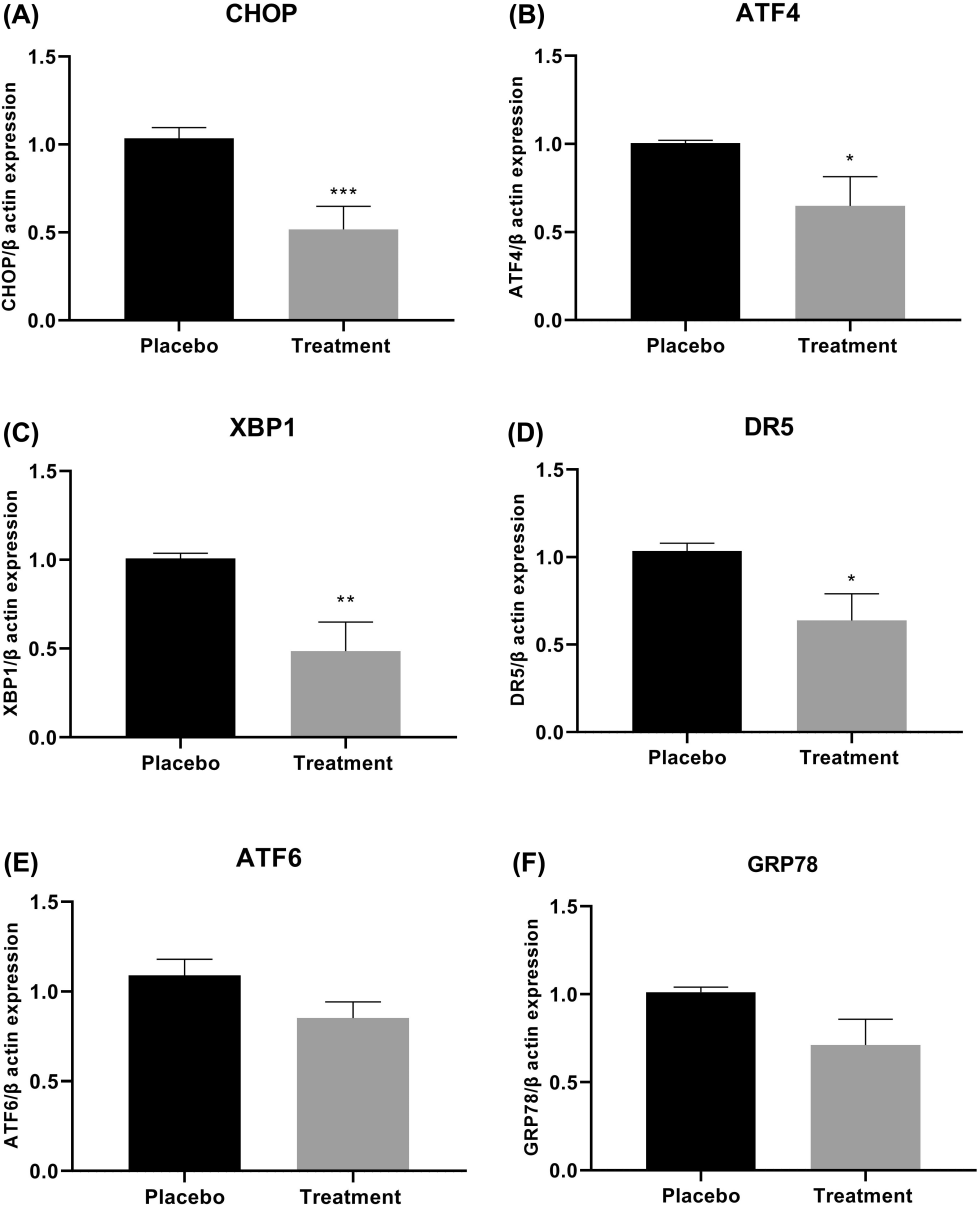
The fold changes levels of CHOP (A), ATF4 (B), XBP1 (C), DR5 (D), ATF6 (E) and GRP78 (F) in pbmcs of placebo and treatment groups. Statistical significance (*p* < 0.05) was assessed by *t*‐test. Differences between groups; *p < 0.05, **p < 0.01 and ***p < 0.001.

**FIGURE 3:**
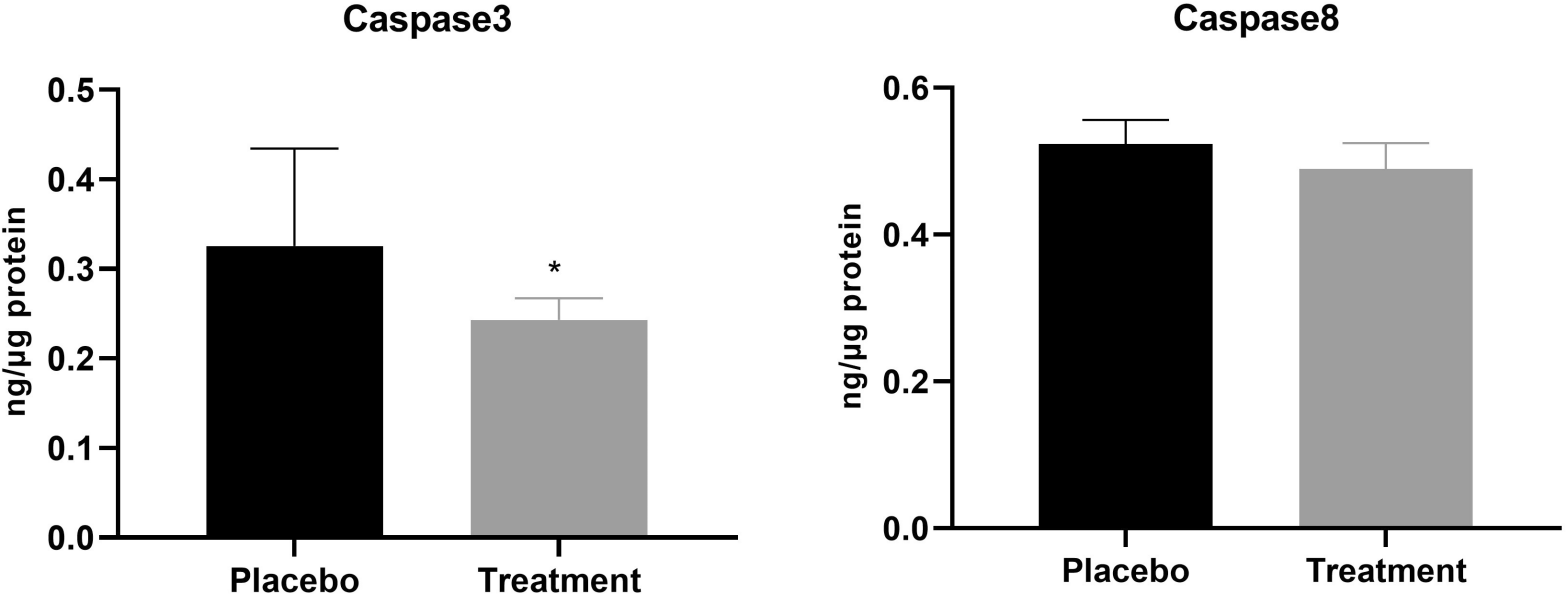
Protein concentrations of active caspase‐3 and caspase‐8 measured using ELISA in PBMCs of placebo and treatment groups. Statistical significance (*p* < 0.05) was assessed by *t*‐test. Differences between groups; *p < 0.05.

In this randomized clinical trial (RCT), 56 participants were first split into two groups of 28. Ultimately, 53 participants completed the trial: 26 in the ASX group and 27 in the placebo group; all dropped out in both groups was for personal reasons (1 and 2 patients in ASX and placebo groups, respectively) (Figure 1). The compliance rate in our study was high, with more than 90% of capsules consumed in both groups during the study. Throughout the trial, no adverse effects were noted in association with the ASX supplement in PCOS women. There were no significant differences between the ASX group and placebo in terms of age, BMI, basal hormones (LH, FSH and Testosterone), disease duration, smoking and alcohol consumption (*p* > 0.05) (Table 2); this also applies to intake of nutrients (Table 3). About physical activity, all of the patients in our study engaged in minimally active category. In terms of inflammatory cytokines, IL‐18, IL‐6, TNF‐α and CRP, there were no statistically significant differences between the two groups at the baseline (*p* > 0.05) (Table 4). Table 4 indicated that, ASX treatment was related to a significant drop in IL‐18 (*p* = 0.003), IL‐6 (*p* = 0.013) and TNF‐α (*p* = 0.009) versus to placebo. In addition, there was no discernible effect of ASX supplementation on CRP level (*p* = 0.177), and BMI (*p* = 0.571) (Tables 2 and 3). In the treatment group, CHOP (*p* = 0.0008; Figure 2A), ATF4 (*p* = 0.038; Figure 2B), XBP1 (*p* = 0.002; Figure 2C) and DR5 (*p* = 0.016; Figure 2D) gene expression levels were considerably lower than the placebo group. Furthermore, the treatment group had lower expression levels of ATF6 (*p* = 0.067; Figure 2E) and GRP78 (*p* = 0.051; Figure 2F) compared to placebo group, but these differences were not statistically significant for ATF6 and only marginally significant for GRP78. Additionally, there was a reduction in the levels of active caspase‐3 (*p* = 0.012) and caspase‐8 (*p* = 0.491) in the treatment group; however, this reduction was statistically significant only in caspase‐3 (Figure 3).

**TABLE 2:** Baseline characteristics of study participants.

Variables	Mean ± SD placebo (*n* = 27)	Mean ± SD intervention (*n* = 26)	*p*‐Values
Age (year)	31.19 ± 4.57	30.42 ± 4.69	0.552^a^
Smoking (yes)	3 (11.11)	0	0.235^b^
Alcohol consumption (yes)	1 (3.7)	3 (11.53)	0.350^b^
Disease duration (year)	4.38 ± 1.9	5.11 ± 1.77	0.157^a^
FSH (μIU/mL)	5.48 ± 1.67	4.98 ± 1.16	0.214^a^
LH (μIU/mL)	9.35 ± 3.21	10.59 ± 2.45	0.121^a^
T (ng/mL)	0.43 ± 0.32	0.47 ± 0.31	0.639^a^
BMI (Kg/m^2^)			
Baseline	26.55 ± 1.89	26.08 ± 1.89	0.369^a^
End	26.51 ± 1.76	25.89 ± 2.20–0.19	0.265
Mean changes	−0.04 ± 1.21	±0.48	0.571

*Note*: Statistically significant (*p* < 0.05), percent change in brackets.

Abbreviations: BMI, body mass index; FSH, follicle‐stimulating hormone; LH, luteinizing hormone; T, testosterone.

^a^ Based on independent *t*‐test.

^b^ Based on Chi‐square.

**TABLE 3:** Dietary intake of study participants throughout the study.

Variables	Mean ± SD placebo (*n* = 27)	Mean ± SD intervention (*n* = 26)	*p*‐Values
Energy(kcal/day)			
Baseline	2264 ± 213.7	2319 ± 207.9	0.349
End	2298 ± 178.1	2337 ± 142.3	0.387
Mean changes	34.01 ± 249.6	17.97 ± 231.0	0.809
Carbohydrates (g/day)			
Baseline	300.3 ± 39.0	316.7 ± 69.00	0.289
End	293.2 ± 24.72	292.9 ± 20.26	0.224
Mean changes	20.73 ± 49.44	26.91 ± 83.05	0.742
Protein (g/day)			
Baseline	81.66 ± 11.09	80.35 ± 13.68	0.701
End	82.55 ± 10.78	84.53 ± 9.21	0.476
Mean changes	0.884 ± 13.31	4.184 ± 14.98	0.400
Fat (g/day)			
Baseline	79.47 ± 10.02	77.66 ± 12.54	0.564
End	78.14 ± 12.61	75.94 ± 11.09	0.502
Mean changes	−1.32 ± 9.89	−1.72 ± 11.82	0.894
SFA (g/day)			
Baseline	15.28 ± 2.97	12.13 ± 2.60	0.218
End	14.22 ± 2.55	15.14 ± 2.85	0.221
Mean changes	−1.05 ± 2.39	−1.14 ± 2.33	0.891
PUFA (g/day)			
Baseline	22.17 ± 3.11	21.18 ± 2.98	0.244
End	20.64 ± 3.302	19.71 ± 3.16	0.301
Mean changes	−1.53 ± 1.57	−1.47 ± 1.97	0.902
MUFA (g/day)			
Baseline	25.08 ± 2.52	24.32 ± 3.01	0.323
End	24.79 ± 2.63	24.71 ± 2.65	0.984
Mean changes	−0.28 ± 1.22	0.45 ± 2.15	0.124

*Note*: *p* (based on independent *t*‐test), statistically significant (*p* < 0.05).

Abbreviations: MUFA, mono unsaturated fatty acid; PUFA, poly unsaturated fatty acid; SFA, saturated fatty acid.

**TABLE 4:** Comparison of serum inflammatory markers between the two groups.

Variables	Mean ± SD placebo (*n* = 27)	Mean ± SD intervention (*n* = 26)	*p*‐Values^a^	*p*‐Values^b^
IL18 (pg/mL)				
Baseline	276.9 ± 43.46	287.0 ± 78.22	0.562	0.038^c^
End	285.9 ± 51.01	259.5 ± 63.30	0.099	
Mean changes	9.05 ± 22.07	–27.52 ± 43.92	0.003^c^	
IL6 (pg/mL)				
Baseline	13.25 ± 3.20	14.19 ± 2.90	0.266	0.146
End	12.93 ± 3.00	12.82 ± 2.61	0.885	
Mean changes	–0.31 ± 1.43	–1.37 ± 1.55	0.013^c^	
TNF‐α (pg/mL)				
Baseline	18.81 ± 1.78	19.25 ± 2.57	0.478	0.002^c^
End	18.25 ± 1.60	17.35 ± 1.29	0.029^c^	
Mean changes	–0.56 ± 1.25	–1.89 ± 2.21	0.009^c^	
CRP (mg/L)				
Baseline	2.42 ± 1.08	2.78 ± 1.35	0.290	0.319
End	2.25 ± 0.83	2.39 ± 1.07	0.603	
Mean changes	–0.17 ± 0.62	–0.39 ± 0.54	0.177	

Abbreviations: CRP, C‐reactive protein; IL18, interleukin‐18; IL6, interleukin‐6; TNF‐α, tumour necrosis factor.

^a^ Based on independent *t*‐test.

^b^ Based on linear mixed effects model (the included variables were: basic value of dependent variable, treatment type, Age and BMI).

^c^ Statistically significant.

In Table 5, it is evident that there were no significant differences between the two groups at the baseline in terms of normal menstrual cycle, hirsutism and hair loss. The study found that there was a slight improvement in menstrual cycle irregularity in both the ASX and placebo groups after intervention. However, this improvement was not statistically significant (*p* = 0.386). The results indicated that, normal menstrual cycle increased after ASX therapy in the treatment group compared to the baseline values (*p* = 0.031). In addition, no significant changes were observed in hirsutism (*p* = 0.398) and hair loss (*p* = 0.784) in both groups.

**TABLE 5:** Clinical symptoms of PCOS at baseline and after 2 months of treatment with ASX and placebo.

Variables	Placebo (*n* = 27)	Intervention (*n* = 26)	*p*‐Values^b^
	Number (%)	Number (%)	
Normal menstrual cycle (yes)			
Before intervention	6 (22.22)	4 (15.38)	0.727
After intervention	7 (25.92)	10 (38.46)	0.386
Intra‐group comparison (*p* ^a^)	1.00	0.031*	
Hirsutism (yes)			
Before intervention	8 (29.62)	11 (42.30)	0.398
After intervention	8 (29.62)	11 (42.30)	0.398
Intra‐group comparison (*p* ^a^)	1.00	1.00	
Hair loss (yes)			
Before intervention	10 (37.03)	14 (53.84)	0.274
After intervention	11 (40.74)	12 (46.15)	0.784
Intra‐group comparison (*p* ^a^)	1.00	0.500	

*Note*: Statistically significant * (*p* < 0.05), percent change in brackets.

^a^ Based on Mcnemar test.

^b^ Based on Chi‐square.

Finally, as shown in Table 6, serum levels of IL‐18, IL‐6, TNF‐α and CRP were analysed for correlations. Correlation analysis showed that serum CRP correlated positively with hirsutism (*r*_s_ = 0.343; *p* = 0.012) and hair loss (*r*_s_ = 0.245; *p*= 0.047). However, no significant correlation was shown between other variables.

**TABLE 6:** Correlations between pro‐inflammatory cytokines and Baseline parameters.

	IL‐6		IL‐18		TNF‐α		CRP	
	*r*_s_	*p*	*r*_s_	*p*	*r*_s_	*p*	*r*_s_	*p*
Age (year)	−0.222	0.110	−0.091	0.515	0.185	0.186	0.002	0.989
BMI (kg/m^2^)	−0.114	0.110	−0.061	0.662	0.148	0.289	−0.028	0.840
FSH (μIU/mL)	0.140	0.318	0.124	0.377	−0.074	0.598	−0.199	0.152
LH (μIU/mL)	−0.122	0.384	−0.026	0.854	0.002	0.989	0.079	0.574
T (ng/mL)	0.115	0.414	−0.123	0.379	−0.074	0.600	−0.110	0.431
Disease duration (year)	0.161	0.251	0.080	0.569	−0.041	0.773	0.102	0.468
Normal menstruation cycle	0.164	0.242	−0.022	0.877	0.069	0.626	−0.043	0.762
Hirsutism	0.176	0.207	−0.175	0.210	−0.076	0.588	0.343	0.012*
Hair loss	0.220	0.113	0.106	0.449	0.018	0.900	0.245	0.047*
Smoking	0.146	0.296	0.077	0.582	−0.002	0.990	0.109	0.437
Alcohol consumption	−0.035	0.801	0.044	0.756	0.022	0.876	0.080	0.570

*Note*: *p* (based on Pearson correlation coefficient), statistically significant * (*p* < 0.05).

Abbreviations: BMI, body mass index; FSH, follicle‐stimulating hormone; LH, luteinizing hormone; T, testosterone.

## 4 DISCUSSION

This RCT examines the effects of ASX consumption on the ER stress‐apoptosis pathway and serum inflammatory markers in PCOS women. The result indicated that, in PCOS women, 8 weeks of oral treatment of 12 mg ASX decreased serum levels of TNF‐α, IL‐18, IL‐6 and CRP. In the treatment group mRNA expression levels of CHOP, ATF4, XBP1 and DR5 decreased compared to the placebo group; however, this reduction was not statistically significant in ATF6, and it also had a borderline effect on GRP78. Also, ASX had no significant positive effect on BMI, hirsutism, hair loss and regularity of the menstrual cycle in women with PCOS. A persistent low‐grade inflammation condition has been reported in patients with PCOS, including elevated leukocyte numbers and dysfunction of pro‐inflammatory cytokines.^43^ Cardiovascular disease and Type 2 diabetes mellitus (T2DM) have been associated with this inflammatory condition.^44^ There is a correlation between chronic inflammatory processes and elevated levels of inflammatory cytokines, such as IL‐1β, TNF‐α and IL‐6.^45^, ^46^ In addition, it has been reported that, PCOS has been linked to greater levels of hsCRP, TNF‐α, IL‐6 and IL‐18 compared in healthy controls.^11^, ^12^, ^47^ High levels of IL‐18 in PCOS patients were found to be correlated with insulin sensitivity and obesity.^45^ It has been observed that IL‐18 induces TNF‐α, resulting in the production of IL‐6.^48^ This suggests that higher IL‐6 and TNF‐α levels in PCOS may be associated with IR, HA and obesity.^49^, ^50^, ^51^ IL‐6 and TNFα stimulate the liver to produce CRP.CRP is commonly used to measure chronic low‐grade inflammation in PCOS studies. A growing body of research suggests that CRP is a biomarker of intravascular inflammation and a key indicator of cardiovascular disease.^52^ Elevated levels of CRP may potentially elucidate the heightened susceptibility of some patients with PCOS to the beginning of cardiovascular disease (CVD) at an earlier age.^53^ ASX is a potent antioxidant that effectively inhibits the initiation of inflammation in various biological systems.^54^ According to previous research, ASX has been shown to reduce inflammatory cytokine expression and modify inflammatory signalling pathways.^55^ It has been shown that ASX reduces TNF‐α, IL1, IL6 and IL18 and modulates NF‐kB and JAK/STAT.^56^, ^57^, ^58^, ^59^ Also, according to a recent review, ASX may be a promising treatment option for chronic inflammatory illnesses and may also protect against skin and gastrointestinal ailments and vascular stiffness by controlling inflammation.^55^ Similar to findings from prior studies, our investigation found that the levels of TNF‐α, IL‐18 and IL‐6 reduced dramatically in the therapy group, it was while the reduction in CRP levels was comparable. Wei Xia et al. discovered in a systematic review and meta‐analysis that taking ASX was associated with a decrease in CRP levels. This association was noticed when ASX was administered for at least 12 weeks or at high doses above 12 mg/day.^60^ It appears that if ASX had been supplied at a higher dose and for a longer duration in our trial, the CRP reduction would have been significant.

Excessive ER stress has been implicated in the aetiology of PCOS and is thought to contribute to cell death. Studies have shown that ER stress inhibitors can ameliorate many PCOS symptoms, which may be a novel entry point for treating PCOS.^61^ The ER protein homeostasis is controlled by the UPR mechanism. While in normal settings, the UPR is not engaged, it can be activated in the presence of certain stressors.^62^ GRP78 is an essential component in maintaining the homeostasis of the ER.^39^ During pathological conditions, GRP78 activates the UPR pathway by releasing three ER stress sensors. Due to this, the degree of ER stress is closely correlated with the expression of GRP78.^24^ ATF4 serves as another indicator of ER stress; it has been shown that ATF4 induces CHOP expression under ER stress. CHOP increases levels of pro‐apoptotic molecules such as DR5 in response to extensive or persisted ER stress.^62^ ATF6, as another branch of UPR, stimulates protein folding and degradation pathways associated with the ER in a manner analogous to IRE1‐XBP1.^63^ ER stress, on the other hand, exerts a close relationship with inflammation and OS. It has been demonstrated that all three main UPR pathways regulate the transcriptional program that promotes inflammation through transcription factors like NF‐ΚB and AP‐1.^33^ In the present study, we determined the effect of ASX on ER stress‐apoptosis in the PBMCs of PCOS patients; the results demonstrated the expression levels of CHOP, GRP78, XBP1, ATF4, ATF6 and DR5 mRNA decreased compared to the placebo group. Similar to our findings, Bhuvaneswari et al. found that ASX decreases the levels of ATF6 and PERK, which preserve liver tissue from the harmful effects of a high fat and fructose diet^64^; on the other hand, it has been shown that ASX attenuated brain injury by reducing levels of GRP78 and CHOP.^40^ Recent research by Wang et al. demonstrated that administration of ASX prevents mice from developing ethanol‐induced cardiomyopathy by lowering their levels of PERK, ATF6, ATF4, GRP78 and CHOP.^39^ Similarly, in another study, Selim Demir et al. found that in the testicular TID model, high levels of ER stress cause testicular tissue damage and that ASX prevents this damage by decreasing the level of ER stress.^65^ A number of studies have suggested that DRs and caspase‐8 may contribute to the promotion of apoptosis subsequent to ER stress.^26^, ^27^, ^30^, ^31^ It has been shown that, caspase‐8 has the ability to initiate the activation of caspase‐3 and caspase‐7.^32^ A growing body of evidence supports a strong correlation between the therapeutic effects of ASX and its anti‐apoptotic properties.^66^ In our prior investigation, we demonstrated that the intake of ASX by infertile women with PCOS resulted in improved levels of apoptotic factors in both serum and follicular fluid, as well as modulation in gene and protein expression related to the apoptosis pathway in granulosa cells.^67^ In the present study, in line with recent studies, we found that, the levels of caspase‐8 and caspase‐3 reduced in the therapy group, however this reduction was comparable in caspase‐8. Based on our data, it can be inferred that PCOS patients exhibit decreased expression levels of ER stress‐apoptosis pathway genes when treated with ASX. This suggests that ASX may activate the surviving branches of the UPR in PBMCs. However, given the strong association between ER stress, oxidative stress, apoptosis and inflammation, it may be argued that regulating the ER stress pathway can help to balance apoptotic pathway and improve inflammatory conditions in PCOS patients.

Multiple studies have examined various therapy approaches that have shown promise in reducing symptoms associated with PCOS, including infertility, menstrual cycle regularity, BMI, IR and HA.^68^, ^69^, ^70^ To the best of our knowledge, this is the first study that investigates the effect of ASX supplement as an adjuvant therapy on PCOS symptoms. The results of our study indicated that, ASX improve menstrual cycle irregularity (38.46% vs. 25.92% of patients in ASX and placebo groups, respectively), but the change was not statistically significant (*p* = 0.386). Also, there were no significant changes in hirsutism (*p* = 0.398) and hair loss (*p* = 0.784) in both groups. There may be several factors that contribute to the variations between our study and other studies, including the type and amount of supplement, participant characteristics (such as age, genetics and body measurements), pharmacokinetic factors, the duration of the participants' follow‐up, and the number of participants. From our perspective, the small sample size is a significant limitation of the present study. Perhaps using a larger sample size in the study would have resulted in statistically significant clinical changes.

The results of this investigation suggest that a positive correlation exists between CRP levels and clinical manifestations of HA (hirsutism and hair loss). However, no significant correlation was shown between other variables. A research study by Nehir et al. demonstrated a positive correlation between FAI and CRP, TNF‐α and α‐1 glycoprotein.^71^ Additionally, prior research has shown a link between inflammatory indicators and HA in PCOS.^72^, ^73^ At present, there is no clarity regarding whether inflammation induces HA by stimulating theca cell androgen synthesis, or whether HA itself initiates the inflammatory response. Some research on PCOS patients suggests that androgens can stimulate inflammatory cells and initiate the inflammatory process. On the other hand, some experts believe that chronic inflammation is the primary factor behind androgen production and ovarian dysfunction.^74^

One remarkable component of our study is the inclusion of all PCOS phenotypes, which considerably increases the possibility of generating data that can be generalized to a larger population. However, it is important to acknowledge that the present study does possess several limitations. Initially, there was a lack of objective measures, such as the quantification of ASX levels in serum or plasma, which made it difficult to evaluate patient compliance The present analysis yielded findings that indicate the potential influence of ASX on the modulation of gene expression related to the ER stress pathway. Nevertheless, it is imperative to assess post‐translation alterations and various other aspects. Ultimately, it is conceivable that the comparatively brief duration of the intervention could have played a role in the development of insignificant results. Hence, it is imperative to undertake further study over an extended duration and with larger sample sizes in order to validate our findings.

In conclusion, our findings show that 8 weeks ASX administration in PCOS patients can modulate ER stress‐apoptosis in PBMCs by changing the expression of genes implicated in the UPR process and apoptosis. ASX, on the other hand, reduced serum levels of pro‐inflammatory markers, such as IL‐18, TNF‐α, IL‐6 and CRP in PCOS patients. More extensive research is required to determine the potential role of ASX.

## AUTHOR CONTRIBUTIONS

**Masoome Jabarpour:** Data curation (equal); formal analysis (lead); investigation (lead); methodology (equal); project administration (equal); visualization (equal); writing – original draft (equal); writing – review and editing (equal). **Fardin Amidi:** Conceptualization (lead); funding acquisition (equal); methodology (lead); project administration (equal); resources (equal); software (lead); supervision (lead); validation (equal); writing – review and editing (equal). **Ashraf Aleyasin:** Conceptualization (supporting); project administration (equal); supervision (supporting); visualization (supporting); writing – review and editing (equal). **Maryam Shabani Nashtaei:** Writing – review and editing (equal). **Mojtaba Saedi Marghmaleki:** Writing – review and editing (equal).

## FUNDING INFORMATION

This study was financially supported by the Tehran University of Medical Sciences, Tehran, Iran.

## CONFLICT OF INTEREST STATEMENT

The authors declare no conflicts of interest.

## CONSENT

Not applicable.

## TRIAL REGISTRATION

This study was retrospectively registered on the Iranian website (www.irct.ir) for clinical trial registration (http://www.irct.ir: IRCT20201029049183N2, 12/04/2022).

## Data Availability

The datasets used and analyzed during the present study are available from the corresponding author on reasonable request.
